# Development of a green synchronous spectrofluorimetric technique for simultaneous determination of Montelukast sodium and Bilastine in pharmaceutical formulations

**DOI:** 10.1186/s13065-024-01116-3

**Published:** 2024-01-25

**Authors:** Sayed M. Derayea, Khalid M. Badr El-Din, Ahmed S. Ahmed, Ahmed A. Khorshed, Mohamed Oraby

**Affiliations:** 1 Department of Pharmaceutical Analytical Chemistry, Faculty of Pharmacy, Minia University, Minia, 61519 Egypt; 2 Department of Pharmaceutical Analytical Chemistry, Faculty of Pharmacy, Sohag University, Sohag, 82524 Egypt; 3 Department of Biomedical Engineering, University of Alberta, Edmonton, AB T6G 1H9 Canada

**Keywords:** Bilastine, Montelukast sodium, Synchronous spectrofluorimetry, Pharmaceutical formulation, Content uniformity

## Abstract

For the treatment of rhinitis and asthma, a combination of Montelukast sodium and Bilastine has just been approved. Based on the first derivative of synchronous fluorescence, the current work developed a green, highly accurate, sensitive, and selective spectroscopic approach for estimating Montelukast sodium and Bilastine in pharmaceutical dosage form without previous separation. The selected technique focuses on measuring the synchronized fluorescence of the studied medications at a fixed wavelength range (Δλ) = 110 nm, and using the amplitude of the first derivative's peak at 381 and 324 nm, for quantitative estimation of Montelukast sodium and Bilastine, respectively. The impacts of different factors on the referred drugs' synchronized fluorescence intensity were investigated and adjusted. The calibration plots for were found to be linear over concentration ranges of 50–2000 ng mL^−1^ for Montelukast sodium and 50–1000 ng mL^−1^ for Bilastine. Montelukast sodium and Bilastine have LODs of 16.5 and 10.9 ng mL^−1^, respectively. In addition, LOQs were: 49.9 and 33.0 ng mL^−1^, for both drugs, respectively. The developed method was successfully employed to quantify the two drugs in synthetic tablets mixture and in laboratory prepared mixtures containing varied Montelukast and Bilastine ratios. To compare the results with the published analytical approach, a variance ratio F-test and a student t-test were used, which revealed no significant differences.

## Introduction

**Fig. 1:**
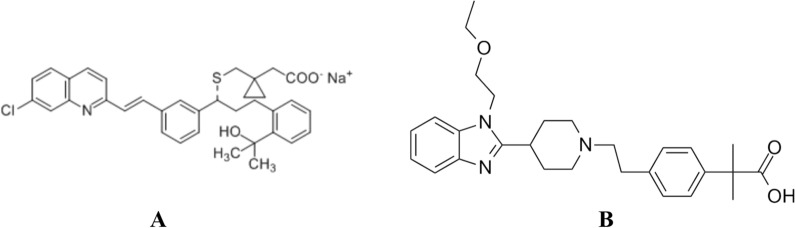
The chemical structure of (**A**): MTK and (**B**): BIL

Allergic rhinitis is a frequent condition that is closely associated with asthma. Asthma was reported in 40% of allergic rhinitis patients, while allergic rhinitis was reported in 30% to 80% of asthmatic patients [1, 2]. A combination of Montelukast sodium (MTK) (10 mg) and Bilastine (BIL) (20 mg) in tablet dosage form has been approved by the central drugs standard control organization in India for allergic rhinitis and mild to moderate asthma therapy. This combination has been highly effective in relieving both rhinitis and asthma [3]. MTK is a cysteinyl leukotriene receptor antagonist used to treat persistent asthma and allergic rhinitis [4, 5]. MTK was approved by the U.S. Food and Drug Administration in 1998 [6]. MTK chemical structure is (R,E)-2-(1-((1-(3-(2-(7-chloroquinolin-2-yl) vinyl) phenyl)-3-(2-(2-hydroxypropan-2-yl) phenyl) propylthio) methyl) cyclopropyl) acetic acid Fig. 1A [7]. The available literature review revealed various methods for analyzing MTK. These methods were spectrophotometric [8, 9], spectrofluorimetric [10, 11], HPTLC [7, 12, 13], HPLC [14, 15] and electrochemical methods [16, 17]. BIL is a second-generation antihistaminic medication that is administered orally to treat allergic rhinitis symptoms [18]. BIL was approved by The European Medicines Agency in September of 2010 [19]. BIL chemical structure is 2-[4-(2-(4-(1-(2-ethoxyethyl)-1H-benzimidazol-2-yl)piperidin-1-yl)ethyl)phenyl]-2-methylpropionic acid Fig. 1B [20]. The published methods for analyzing BIL were spectrophotometric [21, 22], spectrofluorimetric [23, 24], hydrophilic interaction liquid chromatographic method [20], Near-infrared spectroscopy [25], HPLC [26, 27] and electrochemical methods [28].

For simultaneous analysis of MTK and BIL in bulk or pharmaceutical formulations, three different methods were found in the published literature. The methods that have been published for the analysis of MTK and BIL were spectrophotometric [29], HPLC [30] and HPTLC [31, 32]. However, the spectrophotometric method suffered from the low sensitivity as LOQ values for the most sensitive of these methods were 0.36 and 1.17 µg/mL for BIL and MTK, respectively. In addition, the employed wavelength for detecting BIL was very low (110 nm) which make the methods liable for interference even from the diluting solvent. In spite of its high selectivity and appropriate sensitivity, HPLC undergoes several limitations including time-intensive processes for sample preparation and consumed extensive quantities of exceptionally pure organic solvents, which rise both the cost and environmental impacts of the analysis. Although TLC consumed little amount of solvent, the reported method utilized more toxic solvents such as acetonitrile [31] and chloroform [32] and also both methods had limited sensitivity. Therefore, there is a need for a simple and sensitive method for quantifying MTK and BIL concomitantly for quality control purposes which could overcome these limitations. In this regard, synchronous fluorescence spectroscopy (SFS) is among the most sensitive and straightforward methods for analyzing multicomponent samples without the requirement for previous separation. Constant-wavelength SFS is the simplest and most extensively used synchronous fluorescence spectroscopy mode, in which the wavelength interval (Δλ) between both the excitation and emission monochromators is maintained constant [33–35].

Consequently, the major objective of this work is to develop an easy-to-use technique for determining MTK and BIL that complies with green chemistry guidelines and is safe to the environment. In this sense, synchronous spectrofluorometric method combined with first derivative was suggested for the quick, easy, and sensitive determination of MTK and BIL mixtures. The approach did not utilize extreme conditions or dangerous solvents, making the method environmentally friendly.

The proposed technique was designed to meet the standards of the International Council on Harmonization (ICH) [36] and the suggested approach was utilized to determine the drug combination in pharmaceutical preparations and tablet content uniformity.

## Experimental

### Instrumentation

The spectrofluorimetric data were taken using a JASCO FP-8350 spectrofluorimeter (Hachioji, Tokyo, Japan). A 150 W Xe-arc lamp and a PMT set to 400 V power the instrument. For the emission and excitation monochromators, the slit width was 5 nm, and the scanning rate was 1000 nm per minute.

### Materials and reagents

MTK was friendly provided by Hikma Pharmaceuticals (6th of October city, Egypt). BIL was friendly provided by Global Advanced Pharmaceuticals (6th of October, Egypt). Kast® tablets labeled to contain 10 mg of MTK (B.N. 130), were obtained from Hikma Pharmaceuticals (6th of October City, Egypt). Contrahistadin® tablets labeled to contain 20 mg of BIL (B.N. H01702), were obtained from Global Advanced Pharmaceuticals (6th of October, Egypt).

Spectroscopic grade ethanol, methanol, tween, acetonitrile, sodium carboxymethyl cellulose (CMC Na), β cyclodextrin (β CD), polyvinyl alcohol (PVA) and citric acid, were supplied from Merck (Darmstadt, Germany). Analytical grade polyethylene glycol 400 (PEG 400), PEG 6000, dimethylformamide (DMF), and sodium hydroxide were supplied from Fischer Scientific (Loughborough, U.K). Analytical grade sodium dodecyl sulfate (SDS), acetone, phosphoric, acetic, and sulfuric acid were supplied by El Nasr Pharmaceutical Chemical Co. (Cairo, Egypt). Citric acid, sodium hydroxide, and phosphoric acid were mixed in certain proportions to prepare the Teorell and Stenhagen buffer solution PH (3–10) [37].

### Preparation of standard solution

To prepare the stock standard solutions (40 μg mL^−1^), 10 mg of MTK or BIL were dissolved in 250 mL methanol. Working standard solutions were prepared by diluting the stock standard solutions with methanol.

### Procedures for general assay

Several solutions of standard MTK and BIL were prepared by diluting aliquots of stock standard solutions to give (0.5–20 μg mL^−1^) and (0.5–10 μg mL^−1^) of MTK and BIL, respectively. The aliquots were transferred into two series of 10 mL volumetric flasks. Each flask was completed to the mark with methanol after adding 1.0 mL of Teorell and Stenhagen buffer (pH 4). The fluorescence spectra were scanned in the synchronous mode at a fixed wavelength difference (Δλ between the excitation and emission wavelength = 110 nm). The blank experiment was prepared similarly except using the standard drugs. For each synchronous spectrum, data points = 11 were used to record the first derivative synchronous spectra. The peak amplitudes at 381 and 324 nm were used as the analytical signals for MTK and BIL, respectively. The amplitudes of each drug were plotted against their concentration in ng mL^−1^.

### Procedure for laboratory-made mixtures

Different aliquots from the standard solutions (40 μg mL^−1^) were transferred into a series of 10 mL volumetric flasks, to prepare different mixtures of MTK and BIL (1:1, 1:2, 1:3, 1:4, 2:3, 3:2, 4:1, 3:1, 2:1). Teorell and Stenhagen buffer (1.0 mL of pH 4) was added to each flask and was thoroughly mixed. Then the volume was completed to the mark with methanol and the general assay procedure was applied. The concentration of each drug was calculated using the corresponding regression equation.

### Procedure for laboratory-made tablet formulation

The combined dosage form (tablets) of MTK and BIL is not available in Egypt, so combined pharmaceutical tablets were prepared in the laboratory as follows. The contents of 20 MTK tablets (Kast® 10 mg/ tablet) were weighed and the mean weight was calculated. The contents of 20 BIL (Contrahistadin® 20 mg/ tablet) tablets were also weighed and the mean weight was calculated. An amount of the powdered tablet equivalent to 100 mg MTK and 200 mg BIL was weighed and dissolved in 70 mL of methanol. The mixture were transferred to a 100 ml volumetric flask, and mixed well. The content of the flask was sonicated for 15 min, and the volume was completed to the mark with methanol. After that, the solution was filtered and the first portion of the filtrate was discarded. The general assay procedure was applied to a portion of the filtrate using five replicates.

### Procedure for content uniformity test

According to USP guidelines (Chapter 905), a content uniformity (CU) test for MTK and BIL tablets was carried out in this study [38]. A separate analysis of ten Kast® 10 mg tablets and 10 Contrahistadin® 20 mg tablets was carried out with the previous procedure that was mentioned under the analysis of pharmaceutical tablets.

## Results and discussion

**Fig. 2:**
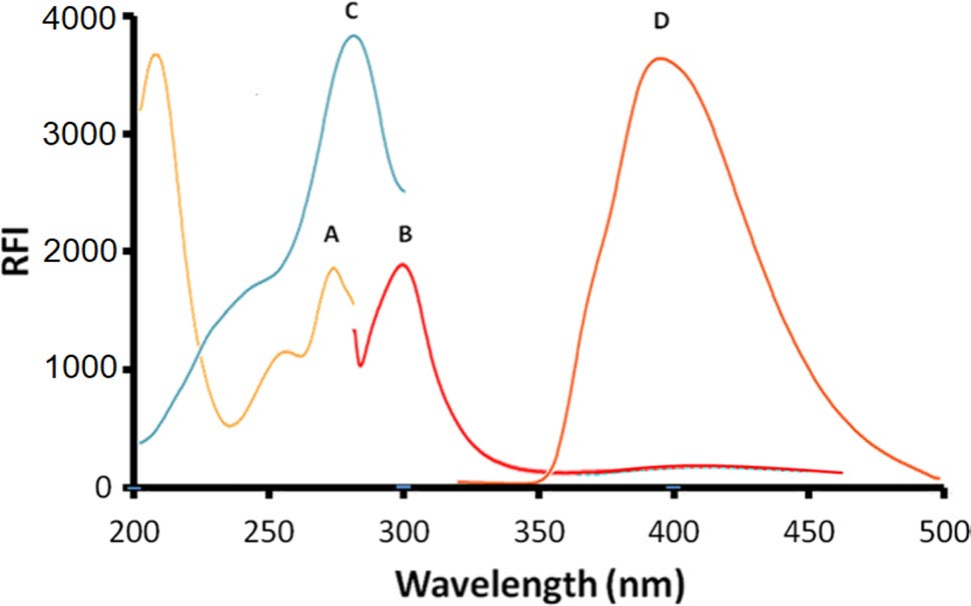
Excitation **(A)**, emission (**B**) spectra of BIL (200 ng mL^−1^), excitation (**C**) and emission (**D**) spectra of MTK (1500 ng m L^−1^) in methanol containing Teorell and Stenhagen buffer (pH 4.0)

**Fig. 3:**
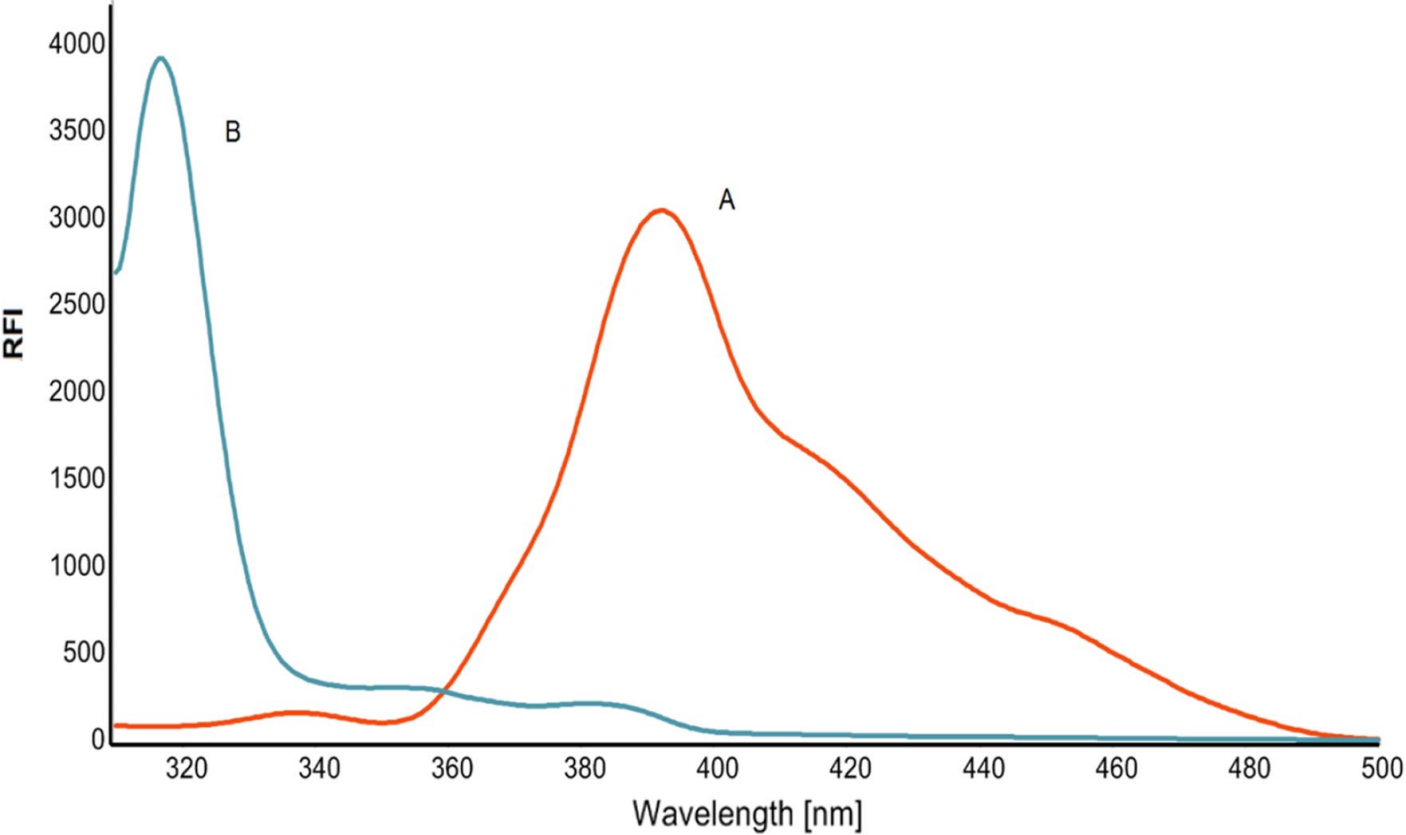
Synchronous fluorescence spectra at delta = 110 nm of: (**A**) MTK 1500 ng mL^−1^ (**B**) BIL 500 ng mL^−1^ in methanol containing Teorell and Stenhagen buffer (pH 4.0)

**Fig. 4:**
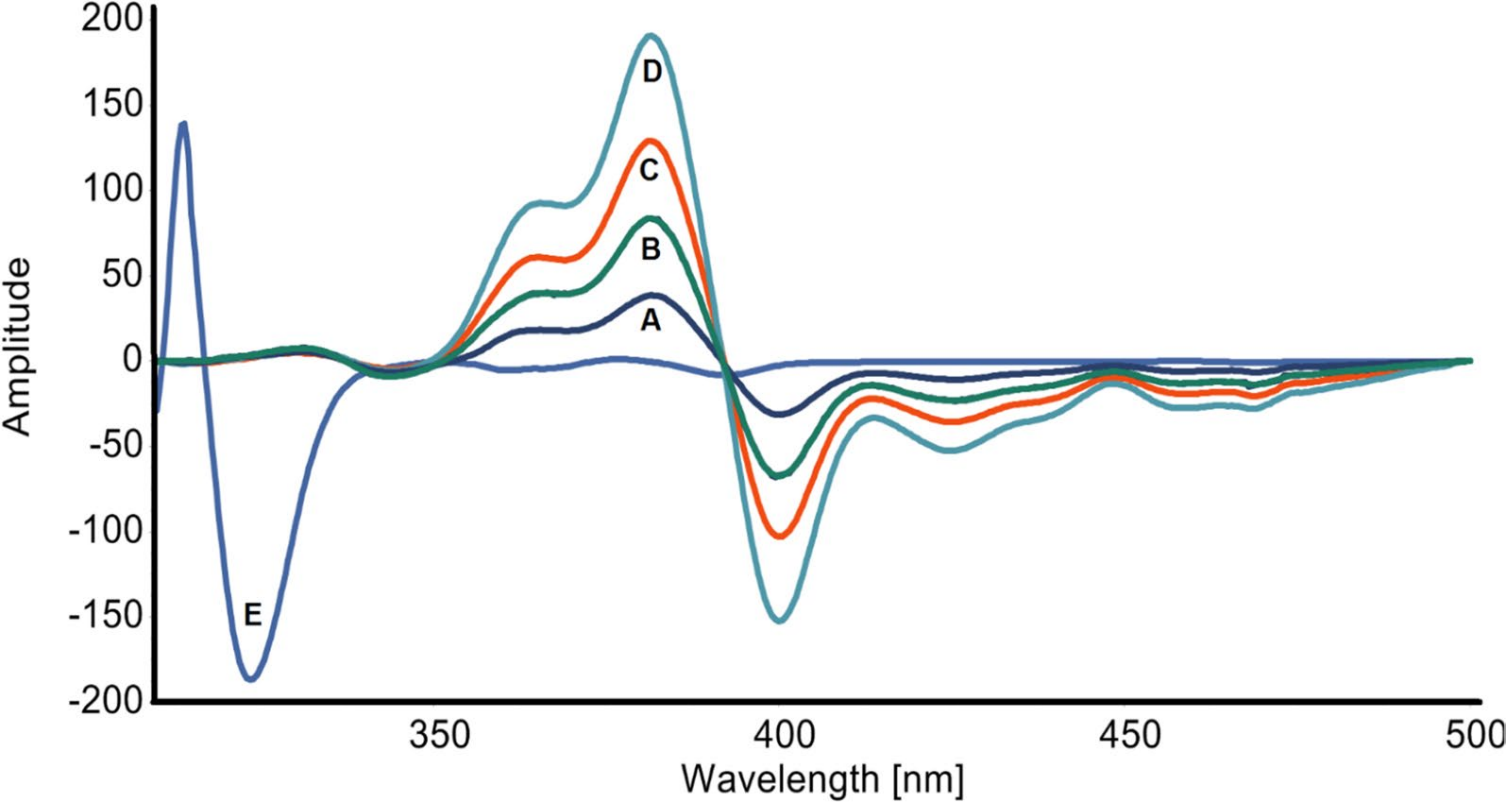
First-order synchronous fluorescence spectra of: (**A–D**) MTK 400, 1000, 1500 and 2000 ng mL^−1^, respectively, and (**E**) BIL 300 ng mL^−1^ in methanol containing Teorell and Stenhagen buffer (pH 4.0)

**Fig. 5:**
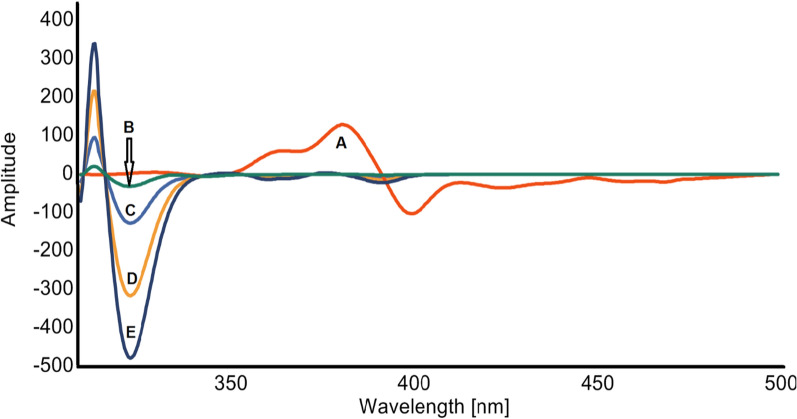
First-order synchronous fluorescence spectra of: (**A**) MTK 1500 ng mL^−1^ and (**B–E**) BIL 50, 200, 500 and 800 ng mL^−1^, respectively in methanol containing Teorell and Stenhagen buffer (pH 4.0)

Constant-wavelength, variety-angle, and constant-energy SFS approaches are categorized based on the distinct scanning modes of monochromators. The constant-wavelength approach, which maintains a constant wavelength difference between the emission and excitation wavelengths, is now the most often utilized method. Fluorescence spectroscopy is known for its exceptional sensitivity. It may enable the researcher to establish detection limits that are significantly lower than those achieved by most of the other analytical approaches. The current study represents the first synchronous spectrofluorimetric method integrated with first derivative for the simple, highly sensitive, and selective quantification of MTK and BIL in bulk powder, and pharmaceutical preparations without prior separation. As shown in Fig. 2, MTK and BIL are fluorescent compounds that release fluorescence at 397 and 298 nm, respectively, after excitation at 285 nm, and 272 nm. However, significant overlapping bands were seen in the their fluorescence spectra, impeding the simultaneous detection of the two drugs by direct spectrofluorimetry. As shown in Fig. 3, measuring the synchronous fluorescence at Δλ 110 nm could decrease the spectral overlap but not completely. Therefore, the first derivative was manipulated for both drugs as shown in Figs. 4, 5. The first derivative synchronous spectra of both drugs were highly separated allowing the feasible determination of MTK and BIL at 381 and 324 nm, respectively, with no interference.

### Optimization of the experimental condition

Different experimental parameters influencing the performance of the suggested method were investigated and optimized. These variables included Δλ, diluting solvents, buffer pH, and surfactant type.

#### Selection of optimum Δλ

The shape, resolution, and intensity of the SFS are all affected by the scanning method. As a consequence, many values (20, 30, 40, 50, 60, 70, 80, 100, 110, and 120 nm) were employed. The best result was obtained with 110 nm resulting in good resolution, consistent peak shape, and the highest fluorescence intensity for both drugs.

#### Effect of diluting solvent

**Fig. 6:**
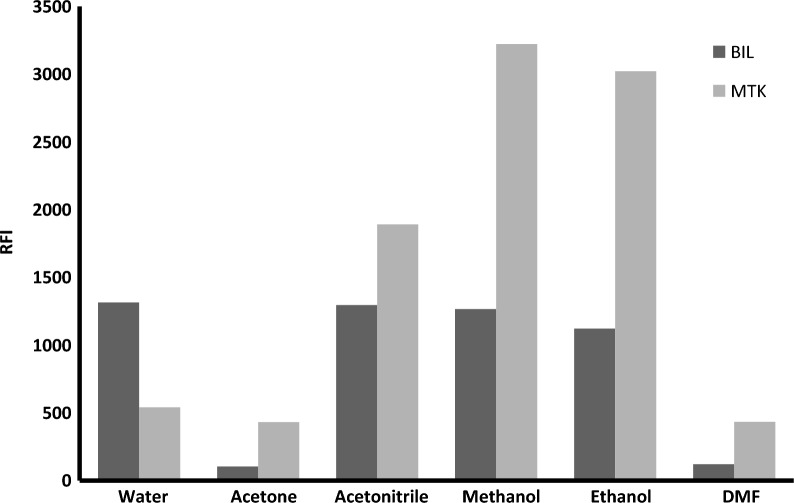
Effect of diluting solvents on the relative fluorescence intensity of 1200 ng mL^−1^ MTK and 250 ng mL^−1^ BIL in the presence of Teorell and Stenhagen buffer (pH 4)

Methanol, water, acetone, ethanol, acetonitrile, and DMF were used to choose the most suitable diluting solvent for MTK and BIL Fig. 6. It was found that; acetone and DMF reduced greatly the synchronized fluorescence intensity, while acetonitrile slightly enhanced the obtained results. The use of ethanol greatly improved the intensity. Meanwhile, methanol produced the highest synchronized fluorescence intensities, so it was used as the diluting solvent for the subsequent work.

#### Effect of buffers and pH modifier

**Fig. 7:**
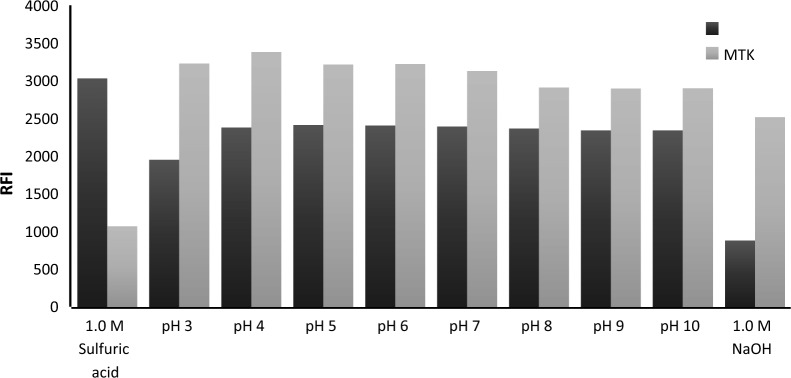
Effect of Teorell and Stenhagen pH, 1.0 M sulfuric acid, and 1.0 M sodium hydroxide on the relative fluorescence intensity of 1200 ng mL^−1^ MTK and 250 ng mL^−1^ BIL

The influence of pH was studied using Teorell and Stenhagen buffer solutions having different pH values (3.0–10.0), in addition to 1.0 M sulfuric and 1.0 M sodium hydroxide as illustrated in Fig. 7. The results indicated that the relative fluorescence intensities of the studied drugs were slightly changed with pH. The relative fluorescence intensity of BIL does not change in a pH ranging from 4.0 to 10.0, while for MTK, a slight increase was observed in the pH range of 3.0–6.0. The use of 1.0 M sulfuric acid greatly enhanced the fluorescence intensity of BIL, but unfortunately, that for MTK was severely reduced. The use of 1.0 M sodium hydroxide decreased the intensity of both drugs. As a result, Teorell and Stenhagen buffer solution (pH 4) was used for the adjustment of the pH as the most appropriate condition for both drugs.

**Fig. 8:**
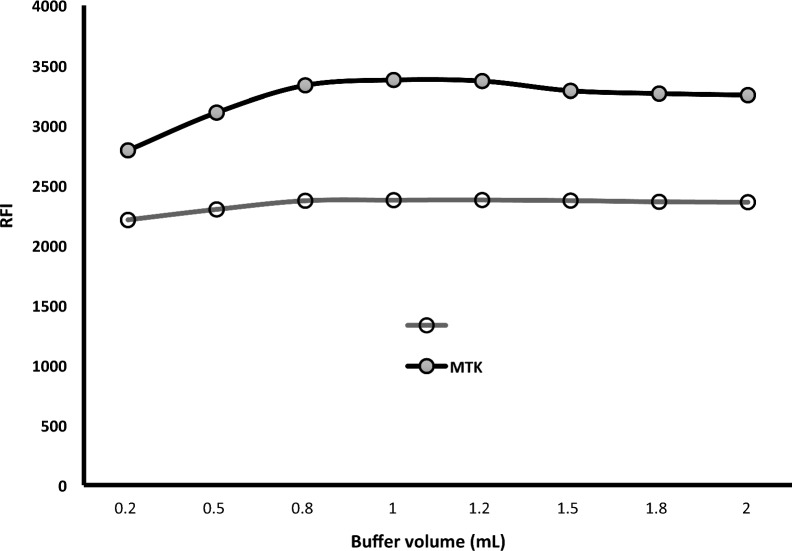
Effect of the volume of Teorell and Stenhagen buffer solution (pH 4.0) on the relative fluorescence intensity of 1200 ng mL^−1^ MTK and 250 ng mL^−1^ BIL

The effect of the volume of Teorell and Stenhagen buffer solution (pH 4) was studied using different volumes in the range of 0.2–2.0 mL. The fluorescence intensity was gradually increased by increasing the buffer volume up to 0.8 mL Fig. 8. A further increase in the buffer volume did not produce any noticeable effect up to 2.0 mL. Therefore, 1.0 mL was suitable for subsequent work.

#### Effect of different organized medium

**Fig. 9:**
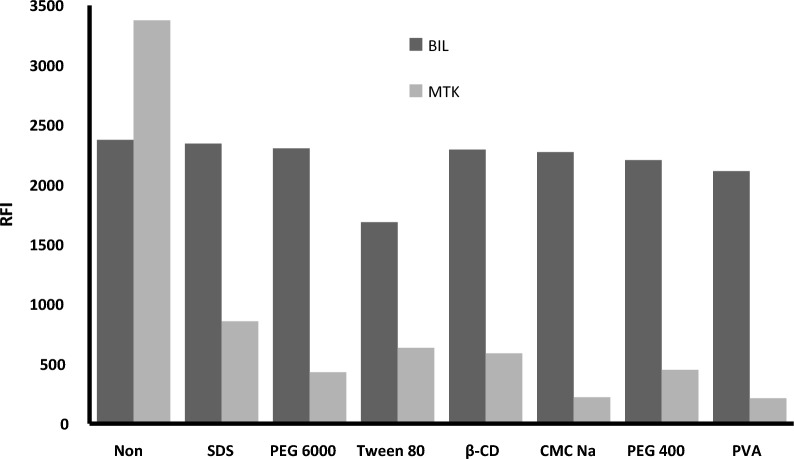
Effect of different surfactants and macromolecules on the relative fluorescence intensity of 1200 ng mL^−1^ MTK and 250 ng mL^−1^ BIL

Different surfactants and macromolecules as organized media were investigated in a trial to improve the fluorescence of the studied drugs. The examined organized media included anionic surfactant (SDS, 0.288% w/v), nonionic surfactant (PEG 6000, 1% w/v, PEG 400, 1% v/v and tween 80, 1% v/v, PVA, 1% w/v), macromolecules (β-CD, 1% w/v), and anionic polysaccharide (CMC Na, 1% w/v) Fig. 9. All the examined substances greatly inhibited the relative fluorescence intensity of MTK. In the case of BIL, the studied substances did not produce any distinct effect except tween 80 which moderately inhibited the fluorescence intensity. Therefore, there was no need for the addition of these organized media.

### Methods validation

The suggested first derivative synchronous fluorescence approach was evaluated and validated using ICH guidelines [36].

#### Linearity and range

The calibration graphs were constructed by plotting the peak amplitude of the first derivative synchronous spectra versus the drug concentrations. For MTK, the linear range was 50–2000 ng mL^−1^, while that for BIL was 50–1000 ng mL^−1^. The correlation coefficients (r) of the regression lines were close to the unity which suggested that the calibration graphs were highly linear. The standard deviation of residuals (S_y/x_), intercept (S_a_), and slope (S_b_) were minimal indicating the low distribution around the calibration lines Table 1.

**Table 1:** The regression and validation parameters for determination of MTK and BIL using the proposed first derivative synchronous spectrofluorometric method

Parameter	MTK	BIL
Linear range (ng mL ^−1^)	50–2000	50–1000
Slope	0.081	− 0.596
SD of slope (S_b_)	0.0003	0.0037
Intercept	2.01	− 5.39
SD of intercept (S_a_)	0.41	0.41
Correlation Coefficient	0.9999	0.9999
SD of residuals (S_y, x_)	2.17	52.59
LOD (ng mL^−1^)	16.5	10.9
LOQ (ng mL^−1^)	49.9	33.0

#### Limit of detection (LOD) and limit of quantification (LOQ)

The ICH guidelines formulae LOD = 3.3 SD/b and LOQ = 10 SD/b (b = slope and SD = intercept standard deviation) were used to calculate the LOD and LOQ. The determined LOD was found to be 49.9 and 33.0 ng mL^−1^ for MTK and BIL, respectively, whereas the calculated LOQ were 16.5 and 10.9 ng mL^−1^ for MTK and BIL, respectively (Table 1). This indicates the higher sensitivity of the proposed method compared to the reported methods [29–32] for the analysis of both MTK and BIL.

#### Accuracy and precision

The method's accuracy was evaluated using the standard addition technique. The obtained results show a high degree of accuracy since the obtained values of % recoveries were close to the real value (100%) as shown in Table 2.

**Table 2:** Accuracy evaluation of the proposed first derivative synchronous spectrofluorometric method using standard addition method on laboratory-made tablet formulation

Amount taken (ng mL^−1^)		Amount added (ng mL^−1^)		Amount found (ng mL^−1^)		% Recovery ± SD^a^	
MTK	BIL	MTK	BIL	MTK	BIL	MTK	BIL
50	100	0	0	50.24	99.89	100.47 ± 0.64	99.90 ± 0.83
50	100	150	100	199.04	200.39	99.52 ± 1.28	100.19 ± 1.32
50	100	750	300	801.59	398.75	100.20 ± 1.31	99.69 ± 1.77
50	100	1350	500	1409.25	604.06	100.66 ± 0.73	100.68 ± 0.39

^a^Mean of five determinations

The precision of the method was assessed by performing the general procedure in triplicate for the determination of three drug concentrations. As seen in Table 3 the values of %RSD were less than 2% which indicated good precision of the method at both repeatability and intermediate precision levels.

**Table 3:** Evaluation of the intra-day and inter-day precision of the proposed first derivative synchronous spectrofluorometric method for the determination of MTK and BIL

MTK Conc. ng mL^−1^	BIL Conc. ng mL^−1^	% Recovery ± RSD^a^			
		Intra-day precision		Inter-day precision	
		MTK	BIL	MTK	BIL
200	200	100.63 ± 0.82	100.95 ± 0.85	100.57 ± 1.17	100.67 ± 0.91
800	400	98.77 ± 0.39	100.96 ± 0.86	99.79 ± 1.37	100.30 ± 1.48
1400	600	99.61 ± 0.32	100.47 ± 0.96	99.97 ± 1.11	100.21 ± 0.97

^a^Mean of five determinations

#### Robustness

The robustness was examined to evaluate the influence of small variations in the experimental parameters on the analytical performance of the proposed method. The examined parameters included pH and the volume of Teorell and Stenhagen buffer. As shown in Table 4 the values of both % recovery and standard deviation were within the permissible limits indicating the reliability of the proposed method during routine use for the determination of BIL and MTK. So, the method is considered robust.

**Table 4:** Robustness assessment of the proposed first derivative synchronous spectrofluorometric method for the determination of MTK and BIL

Parameter	% Recovery ± SD^a^	
	MTK (300 ngmL^−1^)	BIL (600 ngmL^−1^)
Buffer pH		
3.8	99.69 ± 1.14	98.91 ± 1.65
4.2	99.57 ± 1.51	100.13 ± 0.87
Buffer volume		
0.8	99.56 ± 0.87	98.75 ± 0.39
1.2	99.61 ± 1.50	99.33 ± 1.32

^a^The values are the mean of five determinations

#### Selectivity

The effect of different excipients used in tablet formulations was investigated, and the extent of their interference in the results of the proposed method was evaluated. Zinc oxide, talc, lactose, glucose, starch, and magnesium stearate were examined to study the selectivity of the proposed method. Each excipient was added individually to a solution containing a fixed concentration of the studied drugs and the % recovery and standard deviation were estimated. The summarized results in Table 5 showed that these excipients did not affect the selectivity of the suggested method.

**Table 5:** Selectivity evaluation of the proposed first derivative synchronous spectrofluorometric method for the determination of MTK and BIL

Substance added	Amount added	MTK	BIL	% Recovery ± SD^a^	
	μg mL^−1^	ng mL^−1^	ng mL^−1^	MTK	BIL
Talk	10	300	600	99.90 ± 1.27	98.90 ± 1.68
Zinc oxide	10	300	600	100.25 ± 1.67	99.49 ± 0.64
Mg stearate	10	300	600	100.14 ± 1.37	100.06 ± 0.73
Starch	10	300	600	100.06 ± 1.56	98.33 ± 1.03
Glucose	10	300	600	99.95 ± 1.63	99.15 ± 1.30
Lactose	10	300	600	100.54 ± 1.05	100.05 ± 0.50

^a^Mean of five determination

### Pharmaceutical application

The suitability of the developed method for evaluating MTK and BIL in the laboratory-prepared tablet formulation was investigated. The good values of the % recovery that were obtained by the proposed method in Table 6 confirmed the absence of any matrix effect. Indeed, F- and student's *t*-tests were performed to compare the results obtained by the proposed method with that of the reported method [29]. The estimated values of the F- and student's *t*-tests were less than the tabulated values at the 95% confidence limit, indicating that there is no notable difference in precision and accuracy between the proposed and reported procedures. These results prove that the proposed method could simultaneously determine both MTK and BIL in their combined tablets with high accuracy and precision.

**Table 6:** Application of the proposed and reported method for the simultaneous determination of MTK and BIL in laboratory-made tablet formulation

Parameters	Reported method		Proposed method	
	MTK	BIL	MTK	BIL
% Recovery^a^	98.86	99.50	99.05	99.21
Standard deviation, SD	1.08	0.58	0.86	1.14
Number of determinations	5	5	5	5
t-value^a^			0.305	0.507
F-value^a^			1.59	3.85

^a^Tabulated value at 95% confidence limit; t = 2.306 and F = 6.338

### Content uniformity (CU) test application

If the proportion of active elements in the tablet formulation units does not exceed 25% of the total weight of the tablet or if the content of the active constituent is less than 25 mg, it is advised that the CU test of the tablet units should be investigated [38, 39]. Because of its very simple analytical procedure, the presented spectrofluorometric method is ideal for this purpose. For the first time, the spectrofluorometric method was utilized to study the CU of BIL and MTK in their commercial tablets. The active ingredient quantity could be considered uniform in the examined pharmaceutical tablets if the estimated value of AV is lower than or equal to the L1. The AV could be calculated using the following equation:

$${\text{AV}} = {\text{ KS}}\; + \;\left| {{\text{M}}\; - \;\overline{{\text{X}}}} \right|$$

where S represents the standard deviation, K represents the acceptability constant, M represents the reference value, and X̅ is the mean of each tablet’s contents. The obtained AV values using the proposed method for the analysis of Contrahistadin® tablets (20 mg/tablet) of BIL and Kast® (10 mg/tablet) for MTK were less than the L1 value confirming the uniformity of the examined tablet formulations Table 7.

**Table 7:** Application of the proposed method for the content uniformity test of Contrahistadin® and Kast® tablets

Tablet number	% Recovery	
	MTK (Kast® 10 mg)	BIL (Contrahistadin® 20 mg)
1	98.30	100.93
2	96.66	99.89
3	97.18	97.14
4	105.62	97.45
5	99.96	99.82
6	102.95	98.92
7	97.38	99.39
8	100.29	97.08
9	99.36	102.59
10	98.37	104.01
Mean X̅	99.61	99.72
S	2.80	2.30
AV^a^	7.75	6.26
L1^a^	15	15

^a^L1: maximum allowed acceptance value, AV: acceptance value

### Evaluation of method of greenness

Analysts have a significant level of authority when it comes to protecting people and the environment from harmful chemicals and the waste released from industries like chemicals and pharmaceuticals [40]. Green chemistry development and improvement must be carried out repeatedly. The 'ecological value' of the analytical method has been evaluated using certain tools for marking the environmental quality of these methods [41–43]. Three assessment tools were employed to evaluate the environmental friendliness of the current procedure: the Eco-scale [41], the Green Analytical Procedure Index (GAPI) [43], and the Analytical Greenness Calculator (AGREE) [42]. Employing multiple assessment tools is recommended when comparing analytical methods in order to gauge their ecological impact.

In the Eco-scale tool [44] the outcome of the evaluation is a number that represents the penalty points that were issued and subtracted from 100. These issues represent the risks that were faced throughout the research process. The greener the procedure, the higher the score (expressed as a high number). In the present study, methanol was utilized as diluting solvent which has low-toxicity and high flammability. On the other hand, the amount of the waste was very low (≤ 10 mL) and the suggested method consumed less than 0.1 kW/h of energy to analyze a single sample. In addition, the method did not involve heating or an extraction phase. As a result, the suggested method received a 90 on the eco-scale (Table 8), clearly indicating that the present strategy was environmentally friendly.

**Table 8:** Evaluation of the greenness of the proposed synchronous spectrofluorometric method using the eco-scale score approach

Item	Parameter	Word sign	PP sign
Technique	Spectrofluorimetry	LSH	1
Reagent(s)	Non		0
Solvent	Methanol		6
Heating	No heating		0
Temperature	Room tempreture		0
Cooling	No cooling		0
Energy (kW h per sample)	≤ 0.1 KWh/sample		0
Waste	1–10 mL		3
Occupational hazards			0
(TPPs)			10
Eco-scale total score	= 100—TPP		90

LSH for the Less severe hazard, and TPPs for the Total penalty points

**Fig. 10:**
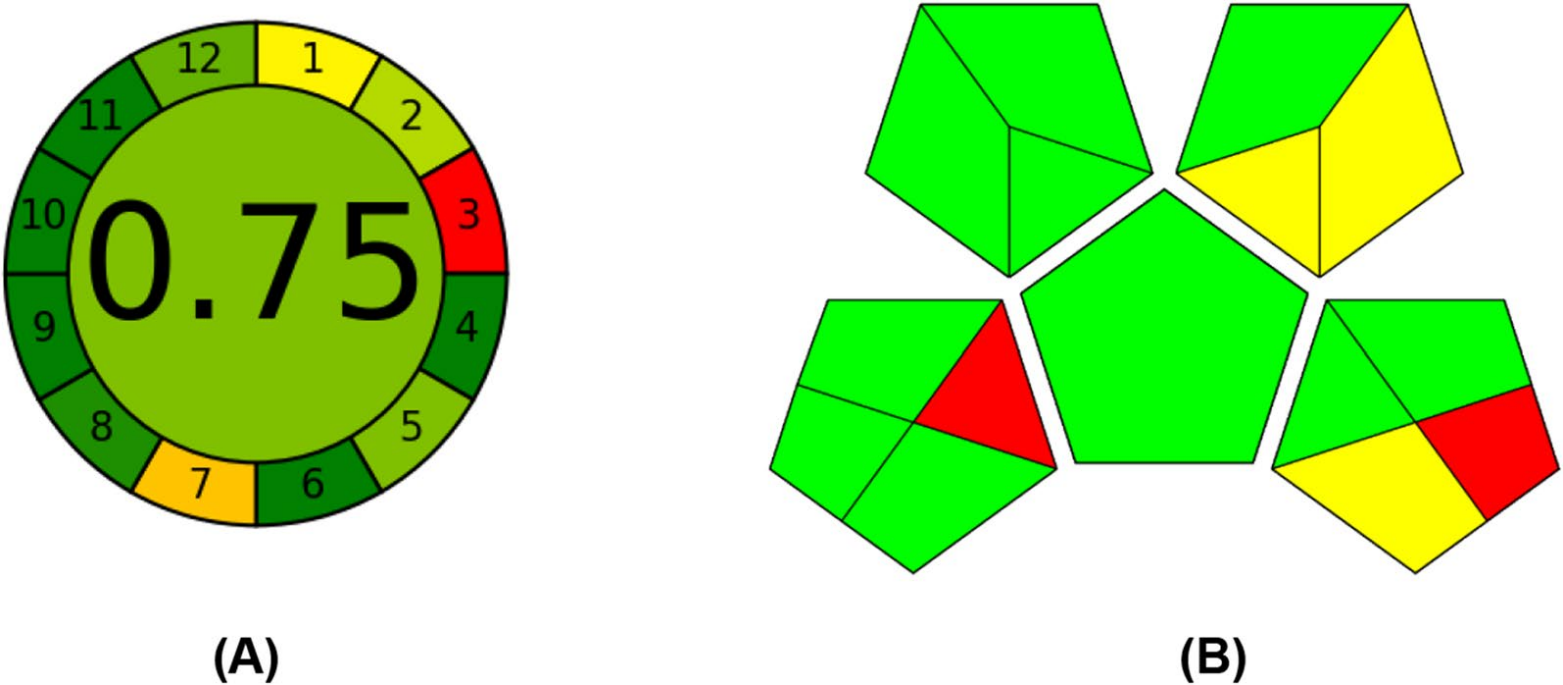
Evaluation of the greenness of the proposed spectrofluorometric method using AGREE (**A**) and GAPI (**B**) methods

GAPI furnishes qualitative data in the form of pictogram symbols [43]. In this metric, a comprehensive assessment of the environmental impact of the analytical procedure was conducted, considering specific details. Five pentagrams were created to assess individual steps in the analytical method that might impact the environment. The evaluation utilized three distinct color codes: green, yellow, and red, representing low, medium, and high environmental impacts, respectively. Illustrated in Fig. 10, the GAPI pentagrams revealed that the current method attains an acceptable green level, as evidenced by 10 green, 2 yellow, and 2 red shaded fields.

Another recently introduced tool for assessing greenness is the Analytical Greenness Calculator (AGREE) [42], offering a straightforward, adaptable, and comprehensive approach. The calculator's software is readily available, and interpreting its results is uncomplicated. The output is presented in pictogram form, with the total score displayed at the center. A score close to one (0.75) and the green color at the pictogram center both signify a high level of greenness in the method. The pictogram also incorporates the twelve principles of Green Analytical Chemistry (GAC), each represented as segments with a color code. Dark green signifies the utmost greenness, while red represents the least green. Figure 10 illustrates the output of the AGREE for the proposed spectrofluorometric method.

## Conclusion

This research developed a very sensitive, green, fully verified first derivative synchronous spectrofluorimetric method for concurrently measuring MTK and BIL. The characteristics of green analytical chemistry were taken into account, with spectrofluorimetry identified for its low energy consumption and utilization of environmentally friendly, less hazardous solvents. The developed method underwent evaluation using three tools; The Analytical Eco-scale, GAPI, and AGREE; and was determined to align with green principles. Furthermore, the spectrofluorometric method stands out for its ease of application, involving straightforward mathematical operations. The developed approach was successful in analyzing both drugs in their laboratory prepared tablets combination without any impact from tablet excipients. This approach is an ideal method for MTK and BIL quality control because of its simplicity and sensitivity as it has lower linear range, LOD, and LOQ than reported analytical methods.

## Data Availability

All data generated or analyzed during this study are included in this published article.
